# Supplementary material for: The Systems Biology Research Tool: evolvable open-source software
Source: BMC Syst Biol. 2008 Jun 29;2:55. doi: 10.1186/1752-0509-2-55 (PMC2446383; doi:10.1186/1752-0509-2-55)
Supplement: Additional file 1 — SBRT Archive. An archive of the current version of the Systems Biology Research Tool. [file 1752-0509-2-55-S1.zip › sbrt-1.4.0/doc/developers_guide/api/sbrt/shell/text/math/LinearCombFormat.html]

LinearCombFormat


|  |  |  |
| --- | --- | --- |
| **Overview** \| **Package** \| **Class** \| **Use** \| **Tree** \| **Deprecated** \| **Index** \| **Help** | |  |
| **PREV CLASS**   **NEXT CLASS** | **FRAMES**    **NO FRAMES**     **All Classes** |  |
| SUMMARY: NESTED \| FIELD \| CONSTR \| METHOD | DETAIL: FIELD \| CONSTR \| METHOD |  |


---


## sbrt.shell.text.math Interface LinearCombFormat<V>

**Type Parameters:**: `V` - the variable type.

**All Superinterfaces:**: Format, Formatter<LinearComb<V>>, MathExprFormat<LinearComb<V>,V>, Parser<LinearComb<V>>, SimpleFormat<LinearComb<V>>

**All Known Implementing Classes:**: LinearCombFormatV1, LinearCombFormatV2

---

``` public interface LinearCombFormat<V> extends MathExprFormat<LinearComb<V>,V> ```

This interface is used to represent formats for linear
combinations.

**Author:**
:   This interface was written and documented by
    Jeremiah Wright while in the Wagner lab.

---

| **Method Summary** | |
| --- | --- |
| `java.lang.String` | `format(LinearComb<V> expression)`             Returns a formatted string representation of the provided linear combination. |
| `LinearComb<V>` | `parse(java.lang.String expression)`             Parses the provided string and returns its corresponding linear combination. |

**Methods inherited from interface sbrt.shell.text.Formatter**

`format`

**Method Detail**

### format

```
java.lang.String format(LinearComb<V> expression)
```

:   Returns a formatted string representation of the
    provided linear combination.

    :   **Specified by:**: `format` in interface `MathExprFormat<LinearComb<V>,V>`
    :   **Parameters:**: `expression` - the linear combination to be formatted. **Returns:**: a formatted string representation of the provided linear combination.

---


### parse

```
LinearComb<V> parse(java.lang.String expression)
```

:   Parses the provided string and returns its
    corresponding linear combination.

    :   **Specified by:**: `parse` in interface `MathExprFormat<LinearComb<V>,V>` **Specified by:**: `parse` in interface `Parser<LinearComb<V>>`
    :   **Parameters:**: `expression` - the string to be parsed. **Returns:**: the linear combination corresponding to the provided string.


---


|  |  |  |
| --- | --- | --- |
| **Overview** \| **Package** \| **Class** \| **Use** \| **Tree** \| **Deprecated** \| **Index** \| **Help** | |  |
| **PREV CLASS**   **NEXT CLASS** | **FRAMES**    **NO FRAMES**     **All Classes** |  |
| SUMMARY: NESTED \| FIELD \| CONSTR \| METHOD | DETAIL: FIELD \| CONSTR \| METHOD |  |


---
